# An initial assessment of the involvement of transglutaminase2 in eosinophilic bronchitis using a disease model developed in C57BL/6 mice

**DOI:** 10.1038/s41598-021-90950-9

**Published:** 2021-06-07

**Authors:** Lan Chen, Shuyan Liu, Linzhuo Xiao, Kanyao Chen, Juanjuan Tang, Chuqin Huang, Wei Luo, Dominique Ferrandon, Kefang Lai, Zi Li

**Affiliations:** 1 Sino-French Hoffmann Institute, Guangzhou, China; 2 State Key Laboratory of Respiratory Disease, Guangzhou Institute of Respiratory Health, Guangzhou Medical University, Guangzhou, China; 3 Université de Strasbourg, M3I UPR9022 du CNRS, 67000 Strasbourg, France

**Keywords:** Inflammatory diseases, Animal disease models

## Abstract

The detailed pathogenesis of eosinophilic bronchitis (EB) remains unclear. Transglutaminase 2 (TG2) has been implicated in many respiratory diseases including asthma. Herein, we aim to assess preliminarily the relationship of TG2 with EB in the context of the development of an appropriate EB model through ovalbumin (OVA) sensitization and challenge in the C57BL/6 mouse strain. Our data lead us to propose a 50 μg dose of OVA challenge as appropriate to establish an EB model in C57BL/6 mice, whereas a challenge with a 400 μg dose of OVA significantly induced asthma. Compared to controls, TG2 is up-regulated in the airway epithelium of EB mice and EB patients. When TG2 activity was inhibited by cystamine treatment, there were no effects on airway responsiveness; in contrast, the lung pathology score and eosinophil counts in bronchoalveolar lavage fluid were significantly increased whereas the cough frequency was significantly decreased. The expression levels of interleukin (IL)-4, IL-13, IL-6, mast cell protease7 and the transient receptor potential (TRP) ankyrin 1 (TRPA1), TRP vanilloid 1 (TRPV1) were significantly decreased. These data open the possibility of an involvement of TG2 in mediating the increased cough frequency in EB through the regulation of TRPA1 and TRPV1 expression. The establishment of an EB model in C57BL/6 mice opens the way for a genetic investigation of the involvement of TG2 and other molecules in this disease using KO mice, which are often generated in the C57BL/6 genetic background.

## Introduction

Eosinophilic bronchitis (EB) was first identified by Gibson and coworkers^1^. It accounts for about 10–30% of Chinese cases of chronic cough, and affects about 10 percent of the adult population in the world^2,3^. EB patients form a group within patients suffering from chronic cough, with eosinophils found in their sputum, but no evidence of airway hyperreactivity (AHR), a defining feature of asthma^4^.The etiology of EB might be linked to the exposure to occupational sensitizers and commonly inhaled allergens^2^. The pathogenesis of EB includes infiltration of inflammatory cells such as eosinophils, Th2/Th17/Treg and mast cells, and the high expression of inflammatory cytokines and mediators, including interleukin (IL)-4, eotaxin, IL-5, IL-13, IL-17A, IFN-γ, IL-1β, IL-6, TNF-α, histamine, leukotrienes, prostaglandin E2 (PGE2), PGD2 and oxidative stress^2,5–17^. The absence of airway hyper-responsiveness in EB patients as compared to asthma patients may be linked to fewer mast cell-infiltrating the airway smooth muscles, relatively lower levels of IL-13 and PGE2 in induced sputum as well as reduced narrowing of the airways^5–9^. More mast cells were recovered from the bronchial brushings of EB patients than those from asthma patients^10^. Anti-IL-5 treatment succeeded in reducing eosinophilic inflammation in asthma and improved airway remodeling, yet was inefficient on cough^18,19^. Therefore, whether eosinophil infiltration causes cough remains an unsettled issue. The interactions between the nervous system and the immune system contribute substantially to the pathogenesis of cough^20–22^.The activated cough receptors, especially the transient receptor potential (TRP) ankyrin 1 (TRPA1) and TRP vanilloid 1 (TRPV1) can stimulate the immune response through acting on mast cells and other immune cells^21^. Mast cells form the bridge between the nervous system and the immune system, and mast cell-superficial nerves interactions might be important in cough associated with eosinophilia^21,23^. Although many researchers have investigated EB’s pathogenic mechanisms, it remains not fully understood at present. It will be important to determine whether a common upstream determinant factor to regulate cough, eosinophilic inflammation and airway resistance exists.

Transglutaminase 2 (TG2 or TGM2) or tissue transglutaminase (tTG) is a ubiquitously expressed member of the transglutaminase family, the members of which catalyze the Ca^2+^-dependent transamidation of proteins. In normal human tissues, it is highly expressed in the placenta, heart, lung, and liver. It is found in several locations within and outside of the cell.TG2 is associated to the plasma membrane, to mitochondria, to the cytosol, to the nucleus and is also found secreted in the extracellular space. It is a multifunctional protein with enzymatic (GTP binding and hydrolysis, protein disulfide isomerase, and protein kinase activities) and non-enzymatic (multiple interactions in protein scaffolds) functions^24–26^. TG2 has been reported to play roles in various cellular processes such as inflammation, apoptosis, development, differentiation, wound healing, and angiogenesis. It is also linked to many diseases such as celiac disease, cancer, tissue fibrosis, diabetes, neurodegenerative diseases^26^. Importantly, it appears to be involved in several lung diseases such as asthma^27,28^, cystic fibrosis^29^, idiopathic pulmonary fibrosis (IPF)^30^, pulmonary hypertension by fibrogenic remodeling^31^, chronic obstructive pulmonary disease (COPD)^32^ and lung cancer^33,34^.

Toluene diisocyanate (TDI) exposure activates TG2 and promotes the production of IgGs against TG2. Airway hyperresponsiveness was significantly lower in TDI-induced occupational asthma subjects with high levels of IgGs against TG2 than those without IgGs against TG2^27^. TG2 regulated the level of IL-13 transcripts and protein in the liver of mice upon *Schistosoma japonicum* infection as well as the levels of leukotrienes (LTs) in bronchoalveolar lavage fluid (BALF) in the OVA-challenged mouse model of asthma^35,36^. Group V phospholipase A2 regulates the TG2 activity of human IL-4-activated M2 macrophages through PGE2 generation^37^. TG2 expression and Ca^2+^ influx are required for mast cell activation in the OVA challenged asthma model. Of note, the high expression level of TG2 in mast cells may contribute to neuroinflammation in Parkinson's disease model^38,39^. The recruitment of eosinophils as well as Th2 and Th17 differentiation were reported to be decreased in OVA challenged TG2-deficient mice compared to WT control^40^. Endothelial cell TG2 is required for allergic inflammation by regulating the recruitment of eosinophils into OVA-challenged lungs^41^. Taken together, these observations suggest the hypothesis that TG2 might be involved in EB, possibly through contributing to the low lung resistance, mast cell related cough sensitivity and eosinophilic inflammation.

Disease models in mice allow the investigation of the pathologic mechanisms of disease. By a sensitization and challenge with 10 μg of ovalbumin (OVA), a model of airway eosinophilic bronchitis without hyperresponsiveness has been set up successfully in BALB/c mice^42–44^. This BALB/c substrain, among other differences, has better reproductive performance (https://www.jax.org/strain/001026). C57BL/6is a more widely used inbred strain and the first to have its genome sequenced. It represents a permissive background for the maximal expression of most mutations (https://www.jax.org/strain/000664). To better identify the pathogenic mechanisms of EB through transgenic mice, the establishment of an EB model in C57BL/6 mice is warranted.

In this study, we initiate the characterization of the relationships between TG2 with EB under the establishment of an EB model in C57BL/6 mice.

## Materials and methods

### Mice, ethics statement, and treatment

**Figure 1:**
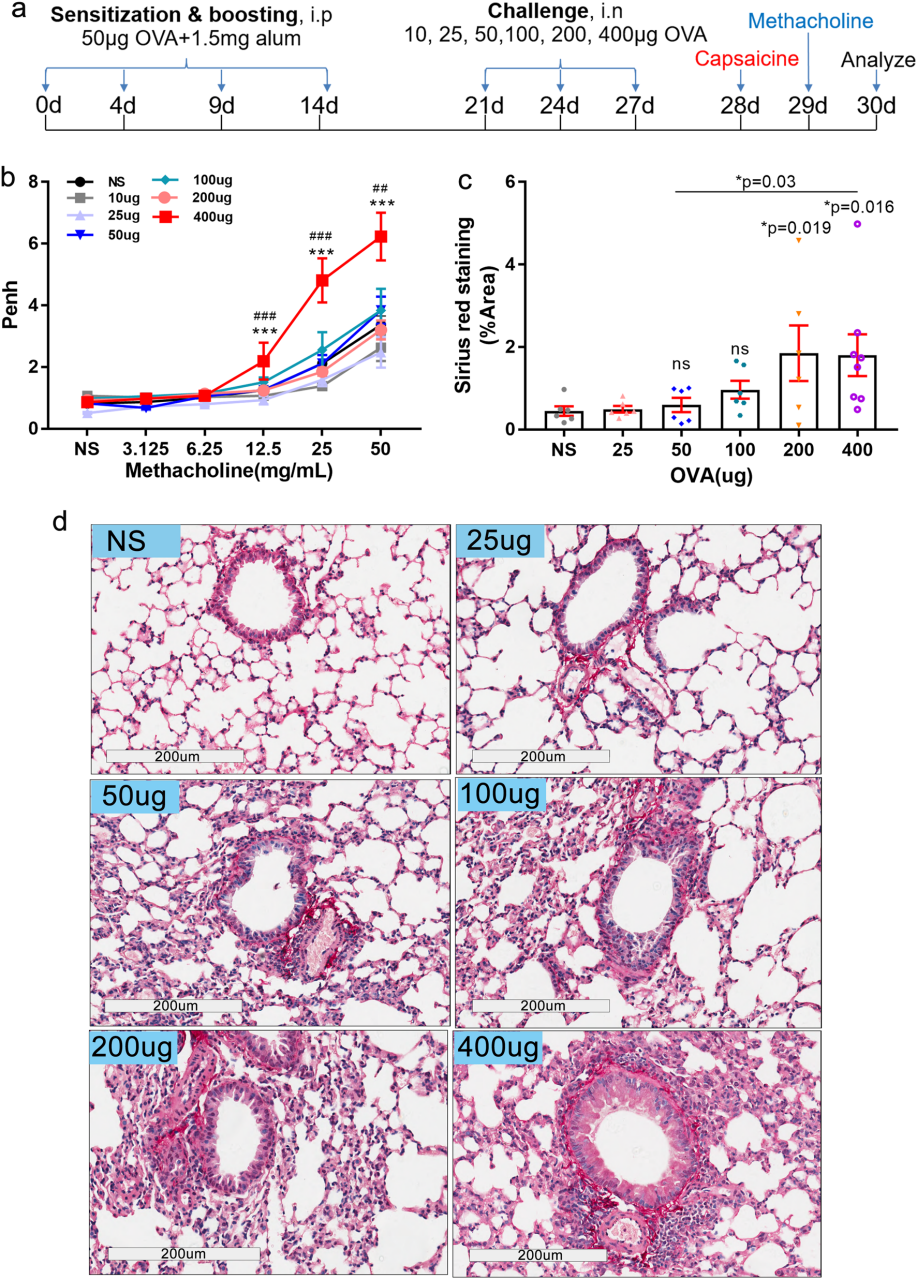
Flow chart of EB model establishment using C57BL/6 mice, lung resistance and collagen deposition measurements. (**a**) Flow chart of EB model establishment using C57BL/6 mice. C57BL/6 mice were sensitized and then boosted with ovalbumin (OVA) plus aluminium adjuvant (alum) intraperitoneally and then intranasally challenged with OVA three times. 12 h after the last challenge, the capsaicine-induced cough frequency was recorded. 36 h after the last challenge, lung resistance or lung enhanced pause (Penh) was evaluated, and finally BALF and lung tissues were subsequently harvested for further analyses. (**b**) Penh in all groups changes in response to increased doses of inhaled methacholine (MCh) (3.125, 6.25, 12.5, 25, and 50 mg/mL). (**c**,**d**) Different doses of OVA challenge affected collagen deposition in lung tissue of mice, as revealed by Sirius red staining. With NS challenge and different doses of OVA challenge, semi-quantitative image analysis of the collagen deposition area (**c**) and representative views (**d**) are shown [25 µg OVA dose, n = 5, other groups, n ≥ 9. *P ≤ 0.05; **P < 0.01; ***P < 0.001; ns, no significance; ^##^*P* < 0.01. *compared with NS, # compared with 50 µg]. The scale is indicated on the panel by the length of the box around the size in µm.

C57BL/6 mice were obtained from Gem Pharmatech (Jiangsu, China). Six to eight-week-old female mice were used for experiments. All animal experiments were performed with the approval of the Institutional Animal Care and Use Committee at Guangzhou Medical University (No. GY2016-0168; GY2017-090). All methods were performed in accordance with the relevant guidelines and regulations. Model building was based on the EB model construction using BABL/c mice and asthma model using C57BL/6 mice^36,37,42–44^. Specifically, mice were administered intraperitoneally 100 μL normal saline (NS) only or 100 μL sensitization liquid [62.5 μL of 50 μg OVA (grade V, Sigma-Aldrich) in NS plus 37.5 μL aluminium adjuvant (Imject™ Alum, 40 mg/mL, Thermo Scientific)] on day 0, 4, 9 and 14. Intranasal challenge of 20 μL NS or NS containing different doses of OVA (10, 25, 50, 100, 200 and 400 μg) were performed on days 21, 24 and 27 (Fig. 1a). During cystamine (CTM) treatment, mice were injected intraperitoneally with CTM (0.01 M, 100 μL each mouse, Sigma-Aldrich) to inhibit TG2 activity (Fig. 6a).

### Airway responsiveness

Lung resistance or lung enhanced pause (Penh) was tested to evaluate airway function after aerosolized methacholine (MCh) (Sigma-Aldrich) inhalation. Mice progressively inhaled 3.125–50 mg/mL MCh for 1 min and the response was measured for 3 min on day 29 (Figs. 1a, 6a). Penh was measured using the BUXCO FinePointe Whole Body Plethysmograph (Data Sciences International, INC., America).

### Measurement of the extent of collagen deposition in mice lung via Sirius red staining

Fresh lung tissues were fixed in 4% paraformaldehyde for 24 h and embedded with paraffin. Lung sections of 4 µm were then prepared for Sirius red staining to evaluate the degree of collagen deposition around bronchiole and semi-quantitative analysis was performed using Image J software analysis.

### Histopathology of lung tissue

Lung tissues were fixed in 4% paraformaldehyde, processed, and embedded in paraffin. Sections were stained with hematoxylin and eosin (HE) staining for lung pathology score analysis and periodic Acid-Schiff (PAS) staining for goblet cell hyperplasia in lung tissue. Lung pathology scoring system according to calibrated criteria (Table 1) was modified from the references^45,46^. Semi-quantitative analysis of PAS staining was performed using Image J software analysis.

**Table 1:** Lung pathology scoring system.

Lung pathology	Score
**(1) No lesions**	0
**(2) Areas of inflammation**	
(a) In peribronchiole or perivascule	1
(b) a + alveolar lumen and alveolar wall	2
(c) b + exudates in bronchi/bronchiolar lumen	3
**(3) Extent of immune cell infiltration including eosinophilic inflammation**	
(a) Some sporadic immune cell infiltration without eosinophils	1
(b) Some immune cell infiltration with few eosinophils	2
(c) Some patchy immune cell foci with apparent eosinophils	3
(d) Multiple immune cell foci with vast eosinophils	4
**(4) Area ratio of exudates in bronchi/bronchiolar lumen**	
(a) Greater than 5% but less than one-third	2
(b) Between one-third and two-thirds	3
(c) Greater than two-thirds	4
**(5) Magnitude of inflammatory foci in the lung**	
(a) Some small patchy inflammatory foci	2
(b) Some large patchy inflammatory foci existed in less than one-third of the whole lung section	3
(c) Confluent inflammatory foci existed in one-third to two-thirds of the whole lung section	4
(d) Confluent inflammatory foci existed in more than two-thirds of the whole lung section	5

### Immune-histochemical (IHC) and Immune-fluorescence (IF) assay of lung tissue

Paraffin-embedded lung tissue sections were deparaffinized and the antigen were repaired using microwave oven heating. Bronchial brushes of EB or control patients were donated with ethical approval from the Ethics Committee of the 1st affiliated Hospital of Guangzhou Medical University (No. 2017-27). All methods were carried out in accordance with relevant guidelines and regulations, and informed consent was obtained from all donors or, if donors are under 18, from a parent and/or legal guardian.TG2 expression level and localization in the lung tissue from EB or control mice with or without CTM treatment, or bronchial brushings from EB or control patients were detected through immunohistochemical assay (IHC). Lung tissues were stained overnight with anti-TG2 (CST, 3557 s) at 4 °C, followed by HRP-conjugated secondary anti-rabbit antibody. Anti-TRPA1(NOVUS, NB110-40763) and Anti-TRPV1(Abcam, ab31895) were incubated overnight at 4 °C on the mice lung sections with or without CTM treatment, subsequently with goat anti-mouse or rabbit cross-adsorbed secondary antibody for 30–40 min at room temperature. The images were observed and captured with an optical microscope (Olympus, Japan) for IHC and fluorescence microscope (ZEISS, Germany) for IF. The semi-quantitative IHC assay was also determined by Image J software and then modified H-score^47^.

### Collection of broncheoalveolar lavage fluid (BALF) and total cells count and cell classification measurements

After measuring lung resistance, 0.4 mL of pre-cooled PBS was injected three times into the lungs via tracheal cannula, and BALF was collected by gradual withdrawal. The standard recovery rate of BALF for each mouse was greater than 80%. BALF was centrifuged 500*g* at 4 °C for 10 min. Supernatant were separated totally and stored at -80 °C for further analysis. The precipitate was resuspended with 100 μL of PBS, and 20 μL for total cell counts using a Neubauer hemocytometer under 200 × microscope, 60 μL for two cytospins. Differential cell counts were measured under a 1000 × microscope (oil immersion lens) after cytospin was stained with Wright-Giemsa. At least 300 cells were counted and further identified as monocytes and macrophages, lymphocytes, neutrophils, or eosinophils according to their standard morphologies.

### Sera IgE concentration measurement using ELISA

Blood was collected from the eyeballs of mice at day 30 and placed at 4 °C overnight, to let the clot form and release the sera. Subsequently, sera were collected, subpackaged and stored at − 80 °C after centrifuged at a speed of 3500 rpm/min for 10 min at 4 °C. IgE concentration in sera from indicated mice were tested according to the instructions of the supplier of the IgE measurement ELISA kit (BD, 555248).

### Determination of cough sensitivity after model establishment

Cough sensitivity in mice was measured by BUXCO FinePointe Whole Body Plethysmograph (Data Sciences Internationl, INC., America) on day 28 (Fig. 1a, Fig. 6a). Specifically, 1 mL of 0.1 mmol/L capsaicin (Sigma-Aldrich) was atomized for 3 min and response for 3 min and then the cough frequences were recorded.

### RNA isolation and quantitative PCR

The total RNA from fresh lung tissues was extracted with Trizol reagent, and cDNA was synthesized using Easy Script® All-in-One First-Strand cDNA Synthesis Super Mix for RT-qPCR (Trans Gen Biotech, China) according to the instructions of the manufacturer. cDNA was amplified by quantitative real-time PCR (qPCR) using the Trans Start® Green qPCR Super Mix (Trans Gen Biotech, China) and the Bio-Rad C1000 Thermal Cycler. Table 2 listed the primer pairs’ sequences. The mRNA level of individual genes was normalized to GAPDH and calculated using the 2^−ΔΔCt^ data analysis method.

**Table 2:** Primer pairs’ sequences of mice genes used in RT-qPCR.

Proteins	Genes	Forward primer sequence	Reverse primer sequence
GAPDH	*Gapdh*	TGTGTCCGTCGTGGATCTGA	TTGCTGTTGAAGTCGCAGGAG
TG2	*Tgm2*	ATCCTGGACCCACTTCTTCTT	CTCTAGGCTGAGACGGTACAG
IFN-γ	*Ifng*	ATGAACGCTACACACTGCATC	ATGAACGCTACACACTGCATC
IL-4	*Il4*	GGTCTCAACCCCCAGCTAGT	GCCGATGATCTCTCTCAAGTGAT
IL-5	*Il5*	GCCGATGATCTCTCTCAAGTGAT	CGGACAGTTTGATTCTTCAGTATG
IL-17A	*Il17a*	GGACTCTCCACCGCAATGAA	TTTCCCTCCGCATTGACACA
IL-9	*Il9*	CTTGTGTCTCTCCGTCCCAAC	ACTATCCTTTTCACCCGATGGA
IL-1α	*Il1a*	ACGTCAAGCAACGGGAAGAT	AAGGTGCTGATCTGGGTTGG
TNF-α	*Tnfa*	CAGGAGGGAGAACAGAAACTCCA	CCTGGTTGGCTGCTTGCTT
IL-6	*Il6*	TGGAGTCACAGAAGGAGTGGCTAA	TCTGACCACAGTGAGGAATGTCCAC
IL-8	*Il8*	TGGGTGAAGGCTACTGTTGG	AGCTTCATTGCCGGTGGAAA
IL-10	*Il10*	GCTGGACAACATACTGCTAACC	ATTTCCGATAAGGCTTGGCAA
COX-2	*COX2*	TTCCAATCCATGTCAAAACCGT	AGTCCGGGTACAGTCACACTT
mPGES-1	*Ptges*	ATTTCCGATAAGGCTTGGCAA	TCACTCCTGTAATACTGGAGGC
Eotaxin	*Eotaxin*	GAATCACCAACAACAGATGCAC	ATCCTGGACCCACTTCTTCTT
MCPT4	*Mcpt4*	TAGACCACATTCTCGCCCTTA	GGATTCTGTCTTGCTCACATCA
MCPT5	*Mcpt5*	GAGATCATTGGAGGCACGGAG	ACTGTTATAGACCTTCCCGCAC
MCPT6	*Mcpt6*	CTGGCTAGTCTGGTGTACTCG	CCAGGGCCACTTACTCTCA
MCPT7	*Mcpt7*	GCCAATGACACCTACTGGATG	GAGCTGTACTCTGACCTTGTTG
CCL2	*Ccl2*	TTAAAAACCTGGATCGGAACCAA	GCATTAGCTTCAGATTTACGGGT
CCL5	*Ccl5*	GCTGCTTTGCCTACCTCTCC	TCGAGTGACAAACACGACTGC
VACM-1	*Vacm1*	GCCCACTAAACGCGAAGGT	ACTGGGTAAATGTCTGGAGCC
SCF	*SCF*	AGCTTGACTACTCTTCTGGACA	TGGCCTCTTCGGAGATTCTTTT
c-KIT	*c-KIT*	GCCTGACGTGCATTGATCC	AGTGGCCTCGGCTTTTTCC
P2X2	*P2rx2*	ACAGAACTGGCACACAAGGG	CAGGGTCATACTTGGGGTCG
P2X3	*P2rx3*	CAGGGTCATACTTGGGGTCG	CGAGCTGATGATGGTGGGAA
TRPA1	*Trpa1*	CGGATGCACACCTCTCCATT	TGCAGGGGCGACTTCTTATC
TRPV1	*Trpv1*	CCGGCTTTTTGGGAAGGGT	GAGACAGGTAGGTCCATCCAC
TRPV4	*Trpv4*	GGACCCTGGCAAGAGTGAAA	AAGCCTGGACTATCTGTGCG

GAPDH, glyceraldehyde-3-phosphate dehydrogenase; IFN, interferon; TNF, tumor necrosis factor; COX, cyclooxygenase; mPGES-1, microsomal prostaglandin E synthase-1; Ptges, prostaglandin E synthase; Mcpt, mast cell protease; VACM-1, vascular cell adhesion molecule-1; SCF, stem cell factor; c-KIT, receptor tyrosine kinase.

### Statistical analysis

Data were expressed as mean ± standard error of the mean (SEM). For comparison of multiple groups, e.g., experiments testing different OVA doses (NS, 10, 25, 50, 100, 200, 400), one-way analysis of variance (ANOVA) followed by least significant difference (LSD) test were performed. The differences between any two groups were evaluated using two-tailed Student’s t-test. All calculations were performed using the GraphPad Prism 7.0 software (GraphPad, San Diego, CA, USA). Differences with *P* < 0.05 were considered statistically significant.

### Ethics approval and consent to participate

C57BL/6 mice were obtained from GemPharmatech (Jiangsu, China). Six to eight-week-old female mice were used for experiments. All animal experiments were performed with the approval of the Institutional Animal Care and Use Committee at Guangzhou Medical University (No. GY2016-0168; GY2017-090). Bronchial brushes of EB or control patients were donated with ethical approval from the Ethics Committee of the 1st affiliated Hospital of Guangzhou Medical University (No. 2017-27). All methods were carried out in accordance with relevant guidelines and regulations, and informed consent was obtained from all donors or, if donors are under 18, from a parent and/or legal guardian. So, the study was carried out in compliance with the ARRIVE guidelines.

## Results

### Intranasal challenge with 50 μg of OVA successfully established an EB model in C57BL/6 mice

OVA-sensitized C57BL/6 mice challenged with different quantities of inhaled OVA antigen were subsequently exposed to aerosolized methacholine (MCh). Mice in the group of a 400 μg dose of OVA challenge demonstrated significantly high lung resistance (also referred to as Penh) after exposure to MCh (3.125–50 mg/mL) compared to mice in the normal saline (NS) group (***P < 0.001, ANOVA followed by LSD-test for each concentration of MCh) or to mice in the 50 μg dose of OVA challenge group (**P < 0.01 or ***P < 0.001, ANOVA followed by LSD-test for each concentration of MCh). Therefore, because a dose of 400 µg of OVA challenge led to a high Penh, this treatment was considered to be a positive control asthma group. Penh in 10, 25, 50, 100 and 200 µg OVA challenge groups was similar to the NS group. The Penh after exposure to these doses of OVA challenge were not significantly different (P > 0.05, ANOVA followed by LSD-test for each concentration of MCh) (Fig. 1b). To evaluate the effects of different doses of OVA challenged mice on collagen deposition in the lung, Sirius red staining was used. A thick collagen deposition was observed around the terminal bronchus of the lungs of mice treated with 400 µg of OVA challenge, in keeping with its high lung resistance (Fig. 1c,d). In conclusion, only the 400 μg dose of OVA challenge group displayed airway hyperreactivity (AHR) and apparent collagen deposition in C57BL/6 mice lungs.

**Figure 2:**
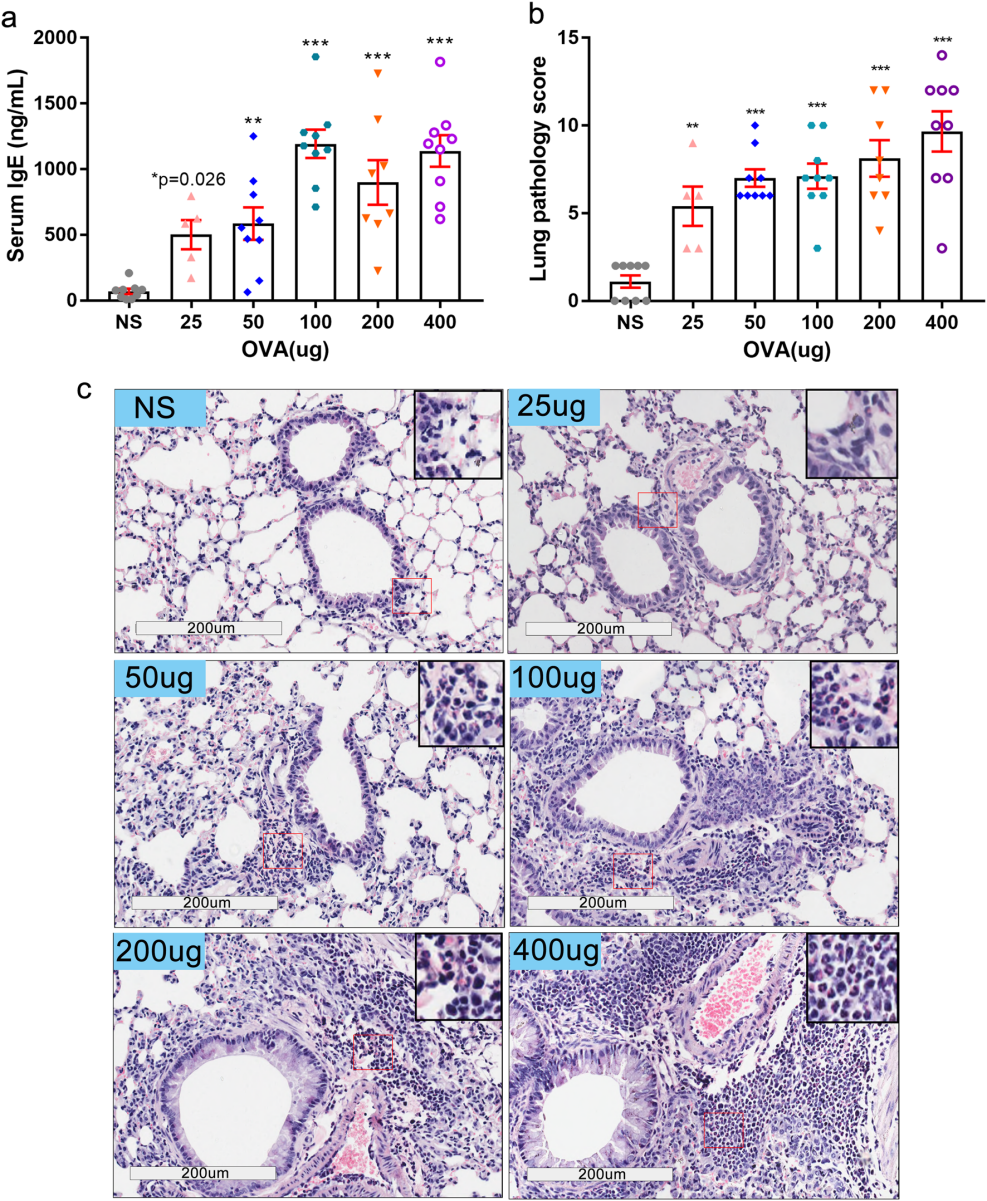
IgE concentration in sera and lung pathology score are significantly increased after OVA intranasal challenges. (**a**) IgE levels in mice sera were all elevated after OVA administration at all doses. (**b**) Lung pathology score according to calibrated criteria (Table 1) from indicated C57BL/6 mice under hematoxylin and eosin staining. (**c**) Representative photographs of (**b**), 200 × , insets show infiltrated eosinophils (red) around terminal bronchioles. (25 µg OVA dose, n = 5, other groups, n ≥ 9. *P ≤ 0.05; **P < 0.01; ***P < 0.001). The scale is indicated on the panel by the length of the box around the size in µm.

The administration of the OVA allergen induced a significant production of IgE at all concentrations as shown in Fig. 2a. The histopathological analysis of lung tissue using HE staining following different doses of OVA challenge revealed that the severity of lung inflammation, scored semi-quantitatively according to criteria shown in Table 1, mirrored the administered OVA dose (Fig. 2b,c). OVA induced the infiltration of inflammatory cells next to the airways.

**Figure 3:**
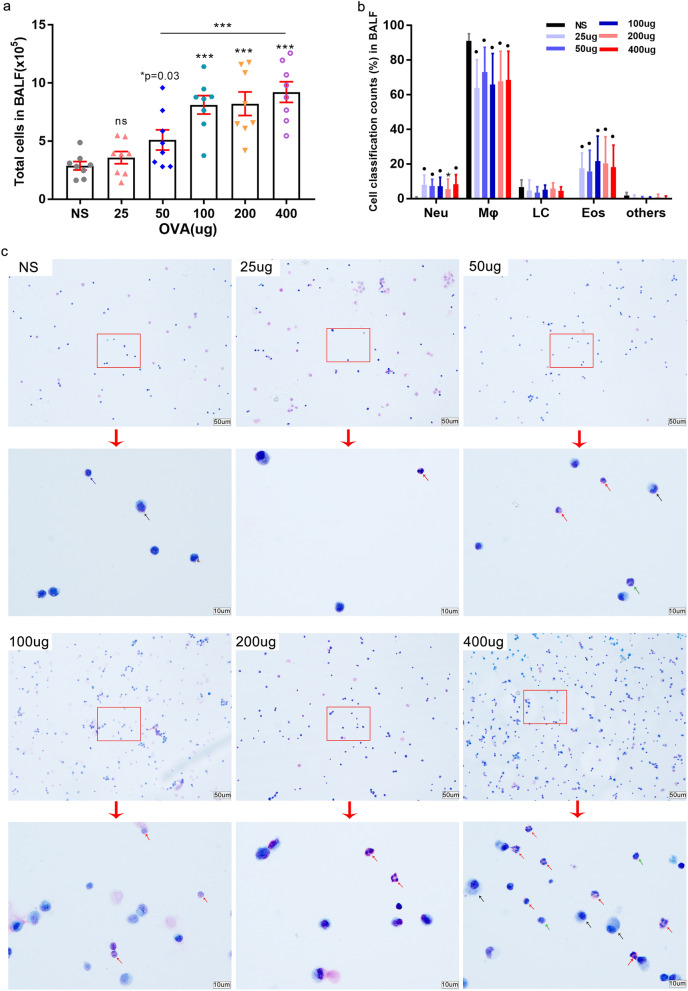
Total cells count and cell classification in BALF after OVA administration. (**a–c**) Bronchoalveolar lavage fluid (BALF) from mice was obtained after airway responsiveness determination. (**a**) Total cells count in BALF were measured using a Neubauer hemocytometer under 200 × microscope. (**b**) Giemsa-Wright stained cells in the BALF were ascribed to distinct categories and counted using a 100 × microscope objective (oil immersion lens) by Wright-Giemsa staining. (**c**) Representative photographs of BALF cells after Wright–Giemsa staining (up, 200 × and down, 1000 × magnifications). Arrows’ colors represent different cells types (green-neutrophils; black-monocytes and macrophages; blue-lymphocytes, red-eosinophils). (25 µg OVA, n = 5; other groups, n ≥ 9. *P ≤ 0.05; **P < 0.01; ***P < 0.001; ns, no significance). The scale is indicated on the panel by the length of the box around the size in µm.

Compared to the NS control group, the total cell count in BALF increased significantly when doses over 50 µg of OVA were used for challenge (Fig. 3a). Cytospin analysis with Wright–Giemsa staining revealed that all OVA-treated mice showed a significant change in the numbers of eosinophils, of macrophages, and of neutrophils, but not of lymphocytes in the BALF (Fig. 3b,c). Eosinophils were observed infiltrated into lung tissue when a dose of 50 μg of OVA was administered intranasally, but fewer eosinophils infiltrated the tissue after a challenge with a 25 μg dose of OVA (Figs. 2c, 3b,c).

**Figure 4:**
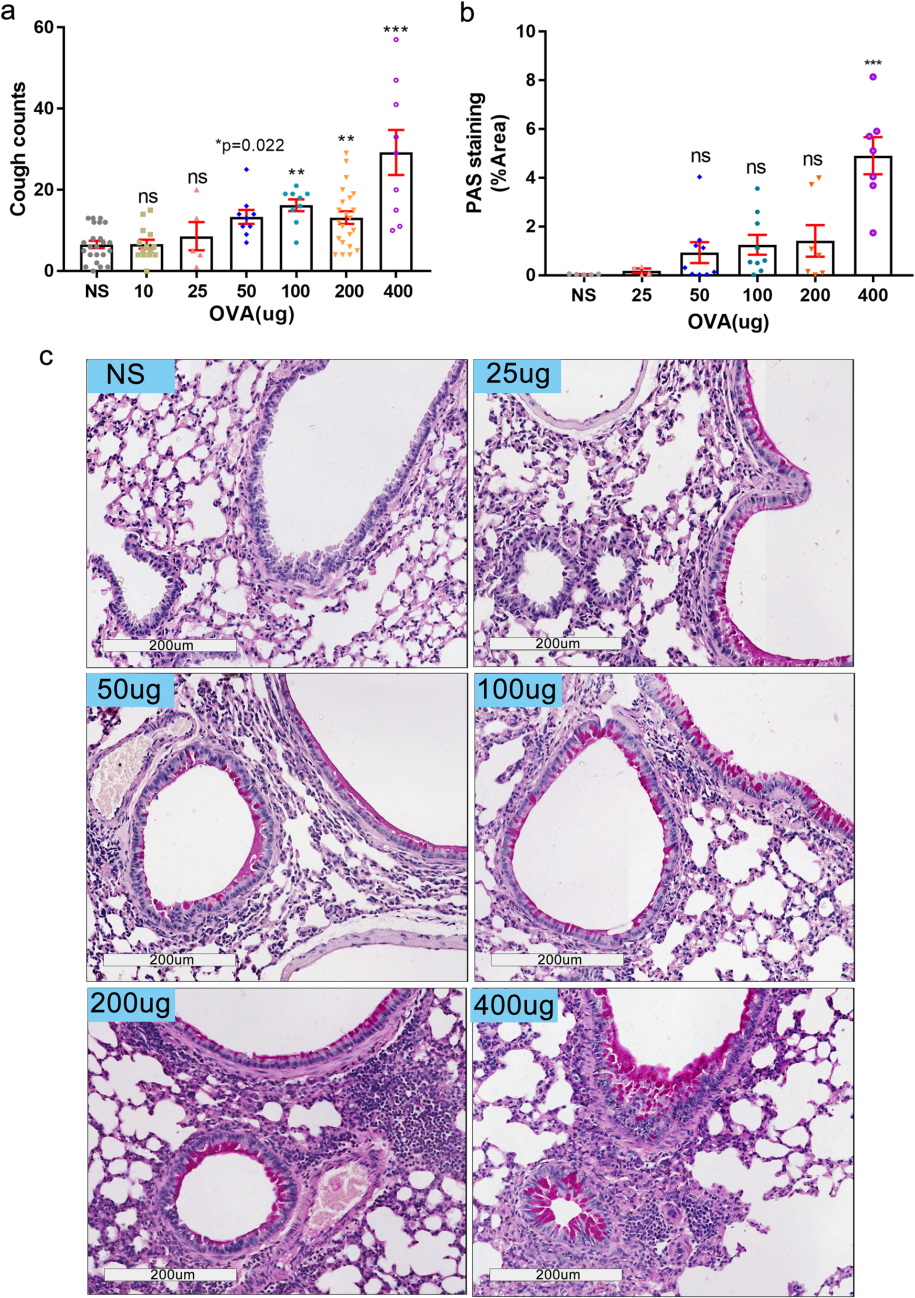
Cough frequency and goblet cell hyperplasia in lung tissue after OVA challenge. (**a**) 12 h after the last OVA challenge, atomized capsaicin (0.1 mmol/L) was used for cough stimulation, and the frequency of cough in mice of all groups was automatically detected using the Finepointe software and recorded. (**b**,**c**) The extent of goblet cell hyperplasia, a significant inflammatory factor leading to wet cough, was estimated by periodic acid-Schiff (PAS) staining. Semi-quantitation of PAS staining was shown as the positive staining area percentage after Image J software analysis (**b**) and representative views were shown in (**c**) (magnification 200 ×). (25 µg OVA dose, n = 5, other groups, n ≥ 9. *P ≤ 0.05; **P < 0.01; ***P < 0.001; ns, no significance). The scale is indicated on the panel by the length of the box around the size in µm.

After NS or OVA challenge, capsaicin was administered to trigger cough. The frequency of coughs was respectively 6.565 ± 0.8887 times for the control group (NS challenge), 6.643 ± 1.097 times for the 10 µg dose of OVA challenge group, 8.6 ± 3.473 times for the 25 µg dose, 12.13 ± 1.253 times for the 50 µg dose, 16.22 ± 1.46 times for the 100 µg dose, 13.18 ± 1.566 times for the 200 µg dose, and 29.22 ± 5.565 times for the group challenged with a 400 µg dose of OVA. A significantly higher cough frequency started from OVA challenge of 50 μg onwards (*P* = 0.022, ANOVA followed by LSD-test) (Fig. 4a). To exclude the possibility of wet cough which characterizes asthma, PAS staining and its semi-quantitative analysis were then used. A significantly high score of positive PAS staining in the terminal bronchus was only observed for the 400 µg OVA challenge group (Fig. 4b,c). This observation suggests that the cough of mice in the 50–200 µg OVA challenge groups is not induced by goblet cell hyperplasia in lung tissue and thus belongs to dry cough category typical of EB.

In summary, a 50 µg dose of OVA challenge is adequate for an EB model using C57BL/6 mice as it does not induce the clinical signs of asthma (no significant high airway resistance nor collagen deposition, low PAS staining extent) while recapitulating the features of EB such as eosinophils infiltration into the lung tissue and in the BALF, higher total cell count in the BALF and increased dry cough frequency.

### TG2 is up-regulated in the airway epithelial cells of EB mice and EB patients

**Figure 5:**
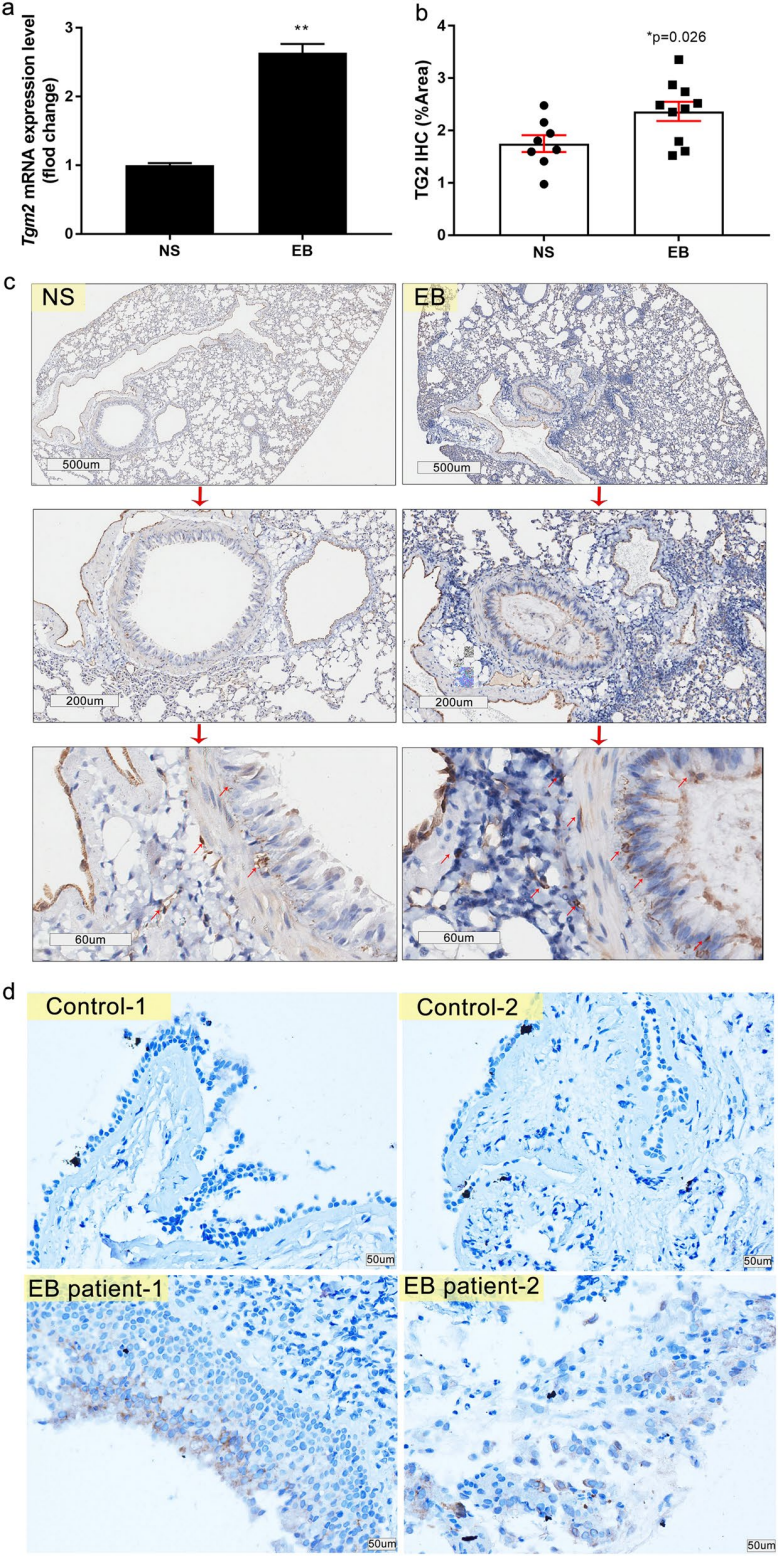
TG2 is up-regulated in the lung of EB mice and in the bronchial epithelial cells of EB patients. (**a–c**) The steady-state mRNA expression levels of *TG2* in mice lung were determined by RT-qPCR (**a**) (figure representative of three dependent experiments) and the semi quantitative level of TG2 protein by immunohistochemistry was quantified (**b**) (n ≥ 8), and typical micrographs are presented (up, 40 × ; middle, 100 × ; and down, 400 × magnification.Arrows showed the location of TG2 positive signal) (**c**). (**d**) TG2 protein expression levels and location were also determined through immunohistochemistry on bronchial brush slices from non-EB control and EB patients (200 × magnification). (*P ≤ 0.05; **P < 0.01). The scale is indicated on the panel by the length of the box around the size in µm.

An involvement of TG2 in lung diseases such as asthma, COPD, IPF, cystic fibrosis and lung cancer has been reported^27–34^. We therefore set out to investigate a connection between TG2 and EB. First, we measured the steady-state transcript levels of *TG2* by RT-qPCR in EB mice at day 30 after challenge and found they were significantly increased (Fig. 5a). Next, we assessed the expression of TG2 in lung tissues by immunohistochemistry (Fig. 5c). The quantification of the antibody signal revealed a significant increase of TG2 detected in histological samples. In keeping with the results obtained in our model of EB in mice, a higher expression level of TG2 was also detected in the epithelium of a bronchus biopsy of two EB patients (Fig. 5d). These results suggest a link between TG2 and EB.

### CTM treatment significantly decreased the cough times but increased the extent of eosinophilic lung inflammation of EB model mice

**Figure 6:**
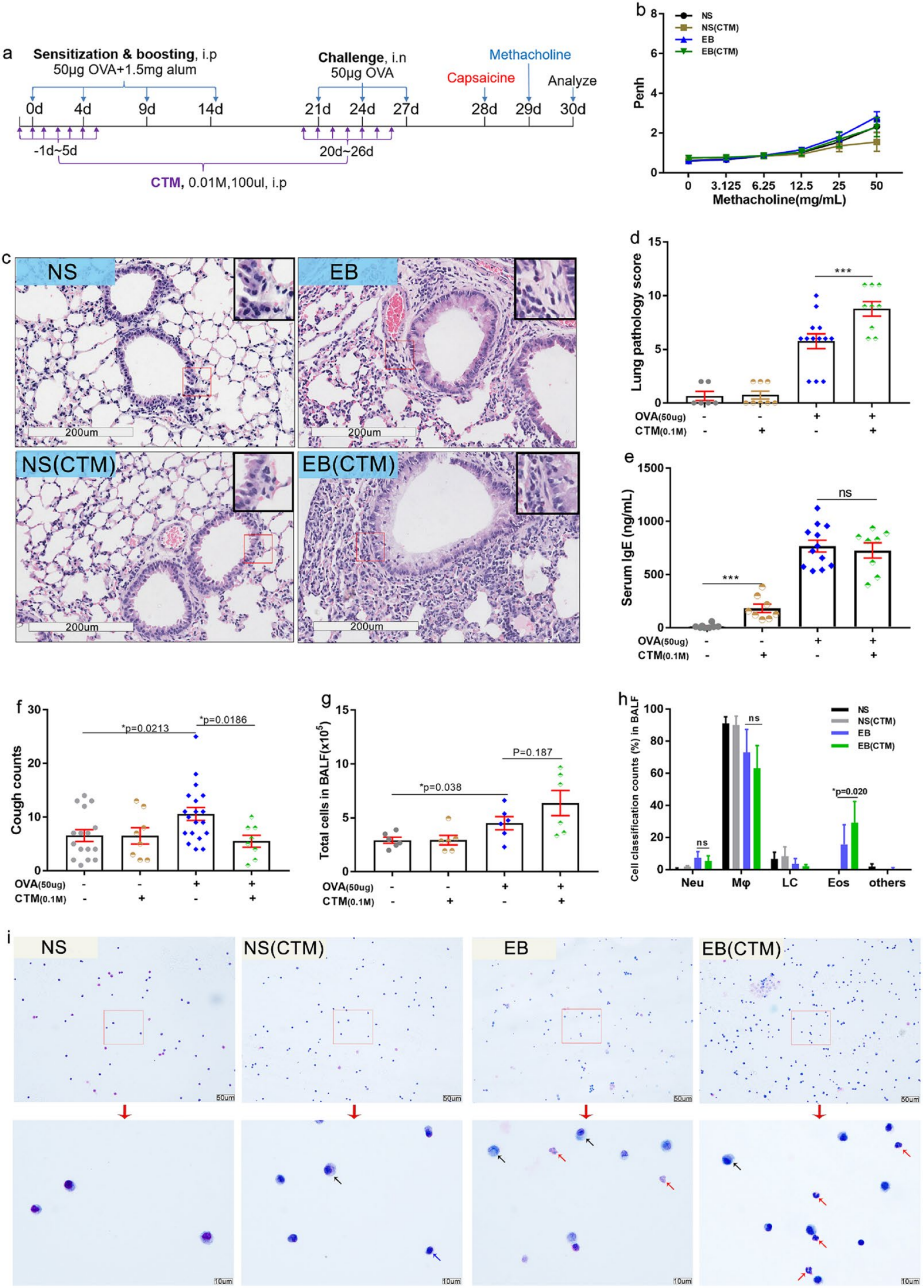
Inhibition of TG2 activity by cystamine treatment lowers the extent of inflammation in the lung and decreases the cough frequency in EB mice. TG2 activity in C57BL/6 mice was inhibited by intraperitoneal injection of CTM (0.01 M, 100 μL each mice) once daily for seven days from one day before the first intraperitoneal OVA sensitization and the first OVA intranasal challenge respectively. (**b**) Penh was evaluated in mice with or without CTM treatment. (**c**,**d**) Lung pathology score of C57BL/6 mice lung sections with or without CTM treatment was evaluated under hematoxylin and eosin staining. Representative photographs (200 × magnification) are shown; insets in (**c**) show eosinophil (red) infiltration around terminal bronchioles. Lung pathology score is displayed in (**d**). IgE concentration in indicated mice sera is represented in **(e)**. (**f**) The frequency of cough in NS or EB model usingC57BL/6 mice with or without CTM treatment was automatically detected using the Finepointe software and recorded. Total cells in the BALF (**g**), differential cells percentage (**h**) and representative photographs of BALF cells’ staining (**i**) were exhibited. (up, 200 × ; down, 1000 × magnification). Arrows’ colors represented different type of cells (black-monocytes and macrophages; blue-lymphocytes, red-eosinophils). (n ≥ 6. *P ≤ 0.05; ***P < 0.001; ns, no significance). The scale is indicated on the panel by the length of the box around the size in µm.

Cystamine (CTM) selectively inhibits the activity of TG2 by modifying the cysteine involved in the disulfide interchange at the active site of TG2. To provide further evidence that TG2 affects the pathogenesis of EB, we treated EB mice by using an intraperitoneal injection of CTM once per day for 7d during OVA sensitization and during OVA challenge respectively (Fig. 6a). After cough frequency and Penh testing, we sacrificed the mice with or without CTM treatment at day 30. Compared to those of untreated mice, Penh measurements were not significantly altered by CTM treatment (Fig. 6b). IgE levels were also not different between the two conditions (Fig. 6e). In contrast, the cough counts of CTM-treated EB mice were significantly lower (Fig. 6f), even though the lung pathology score determined after HE staining was more severe in those mice (Fig. 6c,d). Next, we collected BALF and counted the total cell numbers and identified the different types by Wright-Giemsa staining. Both the number of all cells and eosinophils recovered from the BALF increased upon CTM treatment, although the former was not statistically significant (Fig. 6g–i, insets of Fig. 6c). CTM has been shown to have anti-apoptotic properties^48^ that may be independent from its action on TG2. Thus, these data will need to be confirmed using other TG2 inhibitors such as R2 peptide or TG2 genetic deletion mice. Our data nevertheless suggest that TG2 inhibition can reduce cough frequency.

### Inhibition of TG2 activity lowered the expression of many inflammatory cytokines and cough receptors

**Figure 7:**
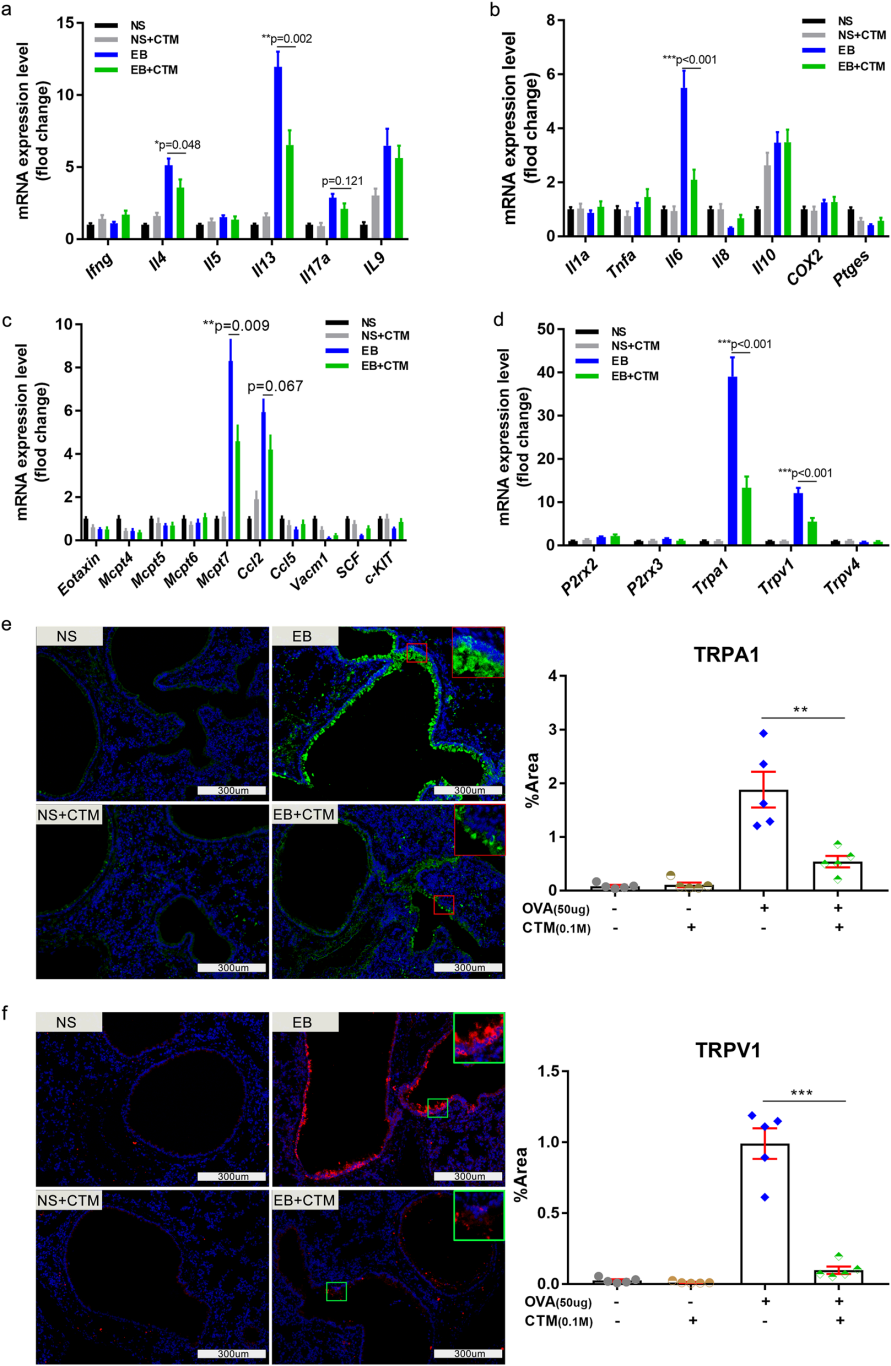
Inhibition of TG2 activity by cystamine treatment significantly lowers the induction of IL-4, IL-13, IL-6, Mcpt7 steady-state mRNA transcripts during EB as well as the induction level of TRPA1 and TRPV1 proteins. (**a–d**) Steady-state mRNA levels in the lung of indicated mice with or without CTM treatment were measured by RT-qPCR. (**a**) Steady-state mRNA levels of IFN-γ, IL-4, IL-5, IL-17A, and IL-9 which are related to Th cell sub-populations. (**b**) Steady-state mRNA levels of IL-1α, TNF-α, IL-6, IL-8, IL-10, COX-2 and mPGES-1. (**c**) Steady-state mRNA levels of eotaxin and some mast cell proteases including MCPT4, MCPT5, MCPT6, MCPT7, mast cell chemokines such as CCL2, CCL5, mast cell adhesion molecular VCAM-1, and mast cell growth factor SCF and its c-KIT receptor. (**d**) Steady-state mRNA levels of some cough receptors including P2X2, P2X3, TRPA1, TRPV1 and TRPV4. (**e–f**) Protein expression level of TRPA1 and TRPV1 with or without CTM treatment were visualized using Immunofluorescence (IF). (**e**) Representative view of TRPA1 (boxes indicate the location of TRPA1 positive staining displayed in the inset) and its semi-quantitation (n = 5). (**f**) Representative view of TRPV1 (boxes indicate the location of TRPV1 positive staining displayed in the inset) and its semi-quantitation using modified H-score (n = 5). The scale is indicated on the panel by the length of the box around the size in µm.

Because of TG2’s possible effect on the extent of eosinophilic inflammation and cough frequency, CTM- treated EB mice were monitored for effects on the transcriptional levels of relevant immune response genes and cough receptor genes. Compared to non-treated mice, the steady-state transcript levels of IL-4, IL-13, IL-6 and Mcpt7 in the lungs of C57BL/6 mice were significantly induced upon OVA-sensitization and re-challenge. Interestingly, this induction was significantly decreased upon CTM treatment (Fig. 7a–c). The transcript levels for IL-9, IL-10, and Ccl2 genes were induced in the EB model but were not altered by CTM treatment. In contrast, the transcription levels of IFN-γ, IL-5, IL-17A, IL-1α, TNF-α, IL-8, COX-2, mPGES-1, eotaxin, Mcpt4-6, CCL5, VACM-1, SCF and c-kit were not altered upon OVA-exposure and re-challenge (Fig. 7a–c).

Cough is a vital protective reflex preventing aspiration into the lung. Noxious stimuli (e.g., gastric fluid, protons, cigarette smoke, particulates, hyper or hypotonicity) induce cough through receptors and ion channels such as P2X2, P2X3, TRPV1, TRPA1, and TRPV4 that are located in vagal afferent nerve terminations of the airway mucosa^4^. The transcription levels of TRPA1 and TRPV1 were significantly increased in the EB model and induced to lower extents upon CTM treatment (Fig. 7d). Consistently, the protein expression level of these two channel proteins were also decreased as TG2 activity inhibition by CTM according to immunofluorescence analysis on lung sections. TRPA1 and TRPV1 expression in the bronchial epithelial cells was induced upon OVA sensitization and re-challenge. However, this induction was much reduced upon CTM treatment (Fig. 7e,f). In summary, TG2 may be involved in stimulating the expression of some cytokine genes as well as that of the TRPA1 and TRPV1 receptors.

## Discussion

In this work, we have provided preliminarily evidence for a role of TG2 in EB by developing an EB model using the C57BL/6 mouse strain by intranasal challenge with a dose of 50 μg OVA. TG2 is upregulated in the airway epithelial cells of EB model mice lung (Fig. 5a–c) and EB patients bronchus (Fig. 5d). Upon inhibition of TG2 activity by CTM, the cough frequency of EB mice was significantly decreased (Fig. 6f). Mechanistically, the induction of the expressions of *IL-6*, *IL-4*, *IL-13*, *Mcpt7*, TRPA1, and TRPV1 in the EB model were significantly reduced upon CTM treatment (Fig. 7). However, the injection of CTM increased the lung pathology score and eosinophils infiltration into the lung of EB mice.

TG2 is a multifunctional enzyme associated with the pathogenesis of many autoimmune, inflammatory, and neurodegenerative diseases^24^. In the airways, increased levels of total TG2 have been observed in induced sputum in asthma^28^, in airway epithelial cells in cystic fibrosis^29^, and in human lung tissue in COPD^32^. In addition, higher TG2 levels were found in lung tissue of non-small cell lung cancer and were linked to poor prognosis for these patients^33,34^. In this work, we aimed to assess whether TG2 might be involved in EB. As expected, TG2 protein expression levels in the airway epithelium of EB mice and patients were increased (Fig. 5). When TG2 activity was inhibited by CTM treatment, the cough frequency in EB model mice was alleviated (Fig. 6g), and consistently the induction levels of *IL-4*, *IL-13*, *IL-6*, *Mcpt7*, TRPA1 and TRPV1 in EB were reduced. Yet, the eosinophil infiltration and lung pathological score were increased (Fig. 7). CTM not only inhibits the activity of TG2, but also displays anti-apoptotic properties^48^. We will further use other TG2 inhibitors such as R2 peptide or TG2 null mice to identify the association of TG2 with eosinophil infiltration and lung inflammation using this EB model in C57BL/6 mice. Indeed, the R2 peptide or TG2 deletion has been shown to reduce eosinophils in BALF in asthmatic mice^36,40^.

Cough has proved to be a troublesome symptom that is refractory to treatments in a subset of subjects. In-depth studies are needed to understand cough physiology and pathophysiology in the hope of identify new targets to treat chronic cough^20^. TRPA1 and TRPV1 have been implicated in the cough reflex sensitization, and they are expressed on afferent sensory airway nerves and airway epithelial cells and are activated by inhaled irritants such as OVA and virus infection. The activated TRPA1 and TRPV1 can stimulate the immune response through acting on mast cells and other immune cells^21,49,50^. Inflammatory cytokines can further reduce the threshold for cough, and stimulate cough receptors directly by acting on airway/lung tissue at sensory nerve endings. A IL-6/TRPV1 & TRPA1 cascade in dorsal root ganglion neurons has been reported to play a role in the development of pain^51,52^. IL-6 released from primary bronchial epithelial cells (PBEC) because of measles virus infection similarly caused TRPA1 and TRPV1 over-expression^53^. It has also been reported that IL-13 up-regulates TRPV1 in the bronchial epithelial cells in the lung of asthmatic BALB/c mice^54^. However, treatment directed at TRPV1 significantly alleviated IL-4, IL-5, and IL-13 level in a chronic asthma murine model, while conversely TRPA1 promoted OVA-induced IL-4 production during acute allergic lung inflammation^55,56^. These findings will guide us in further identifying the relationship and mechanisms of TG2 interaction with TRPA1, TRPV1 and IL-6 & IL-4, IL-13 using TG2 null mice and in vitro cultured cell. On the other hand, mast cells form a bridge between the nervous system and the immune system^23,57^. The activation of mast cells in superficial airway structures is a specific feature of EB ^2,10^. The number of mast cells in bronchial brushing samples and the sputum concentration of mast cell mediators were greater in subjects with EB than in those with asthma^2^. As reported, TG2 expression and Ca^2+^ influx are required for mast cell activation and IgE production from B cells in mouse allergic asthma^38^. Here, in C57BL/6 mice EB, we only tested the transcription level of some chemokines and proteases produced by mast cells and found that TG2 inhibition only down-regulated Mcpt7 level. We will use TG2 genetic deletion mice and in vitro cell co-culture systems to investigate how a TG2-mast cell axis may be involved in EB.

The concept of EB emerged in 1989^1^, and the deep mechanisms’ study of EB relies on the model establishment. In 2011, Chen et al*.* demonstrated for the first time that an EB model in BALB/c mice can be established using a low-dose OVA sensitization and challenge (10 µg)^42,44^. C57BL/6 is the most widely used inbred strain, the first strain to have its genome sequenced, and constitutes a permissive background for the optimal expression of most mutations (https://www.jax.org/strain/000664). It was therefore necessary to establish an EB model in this strain to allow an in-depth study of the pathogenic mechanisms of EB through transgenic mice. Herein, we successfully established an EB model in the C57BL/6 mouse strain by sensitization and then challenge with a dose of 50 μg OVA (Fig. 1a). The model reproduces the main pathological features of EB without inducing an airway hyperreactivity (AHR) (Fig. 1b). The mice in the EB model displayed a significant increase in total cell counts in BALF (Fig. 3b, 3c), apparent eosinophils infiltration into lung tissue (Fig. 2), and a significantly higher cough frequency (Fig. 4a). Only a 400 µg dose of OVA induced AHR, apparent collagen deposition (Fig. 1) and mucin production (Fig. 4b,c) in C57BL/6 mice lung. Therefore, this challenge dose acted as asthma group or positive control for high lung resistance. C57BL/6 mice tend to show type 1 inflammatory responses while BALB/c exhibit a type 2 immune response because of their different genetic backgrounds^58,59^. Both EB and asthma belong to type 2-driven processes^60^. This divergence in immune reactivity likely explains why higher doses of OVA were needed to establish the EB & asthma model in C57BL/6 as compared to the BALB/c strain. In fact, besides the doses for OVA sensitization and challenge, we also had to increase the sensitization and boosting frequency from two or three to four times (Fig. 1a). We have tried two or three rounds of boosting with a 10 µg dose of OVA and then challenges of the mice using 10, 25 or 50 µg doses of OVA and failed to induce the infiltration of eosinophils into the lung, high cough frequency and AHR (data not shown). To evaluate the adequate dose range for OVA challenge to construct the EB model, we examined not only the Penh, but also the collagen deposition using Sirius red staining and the mucin production through PAS staining, which are prominent features of asthma^27,28^. Finally, our strategy of four rounds of sensitization with 50 µg doses of OVA followed by at least 50 µg of OVA re-challenge leads to the induction of clinical signs of EB in C57BL/6 mice.

In conclusion, our findings suggested the enrollment of TG2 in EB possibly through mast cell, inflammation, cough receptor regulation, or the interactions among them. A challenge with a 50 µg dose of OVA intranasal can successfully establish an airway eosinophilic bronchitis model using C57BL/6 mice, without triggering AHR. Future studies will reveal whether the interaction of TG2 with other components involved in EB may constitute a relevant novel therapeutic target to treat EB.
